# Indents

**Published:** 1915-05

**Authors:** 


### INDENTS.

☞ **Brief Articles of Technical or Scientific Value will receive notice and comment. Contributions invited.**

*What can the Unsuccessful Graduate do?* Dental colleges should incorporate in their curriculum a course in public dental education and prophylaxis, and require that their students should become efficient in the presentation of illustrated lectures on this subject, using the faculty and the student body as their audience upon whom to practice. Then if a man failed to pass the state board examination there would be a tremendous field left open to him, one ten times greater, and, if you will allow me to say it, of one hundred times greater importance than if he were commissioned to practice and do technical work for suffering humanity. It seems to me that this would afford the colleges an opportunity to use material which otherwise they could not use. There are young men who can not qualify technically to save aching molars, but who might prove to be geniuses in the field of platform work, and who by lecturing before the thirty millions of public school children in the United States would be instrumental in saving hundreds of thousands of first molars. There is no law so far against a man speaking in public schools on prophylaxis; probably the time will come when we will have licensed men to do that, but at present the field is open. So instead of paying money for a dental equipment, the student might buy a lantern and lantern slides.

This is not a dream. I realize that I am open to the criticism of having been a dreamer all my days, but this is practical common sense, and it seems to me a not unreasonable request to make of the dental colleges of the United States.—J. P. Corley, in *Cosmos*.

*Amalgam and Gold Inlay Restorations.* It is patent to most of us, who have had experience with gold inlays and with amalgam, that a more efficient occlusal surface can be developed with the gold inlay than is possible with amalgam, and I believe it to be equally plain to those who have had wide experience with both materials, that the gold inlay is the better and more efficient protector of the integrity of the all-important root of an extensively broken down molar than is the amalgam restoration.

I am quite ready to accept amalgam as still having a very important place in our procedures. I consider it as standing second only to gold inlays in its efficiency for such restorations, and that it will so continue. Further than this, it would undoubtedly be a greater loss to our profession and to our clientele to be deprived of amalgam than it would to be deprived of gold inlays. More dentists, and many more of their patients would be inconvenienced by its loss.—Dr. H. W. Gillett, in *Journal of Allied Societies.*

*How to Protect Gold Work from Amalgamation.* Before inserting an amalgam filling in a mouth containing gold, especially if the amalgam is in close proximity to a gold filling or crown, dry the gold work and cover it with a coat of sandarach varnish.—A. F. Danahower, *Dental Cosmos.*

*Temporary Stopping for Cavities.* In a sensitive cavity a temporary stopping can be inserted most comfortably by sticking a piece of stopping to the end of a broad, flat burnisher. Then wipe the cavity as dry as possible with a pledget of absorbent cotton, and after heating the gutta-percha over the flame, plunge it into a bottle of oil of cajaput and force it into the cavity. The gutta-percha in this way is cooled, thereby avoiding any shock to the sensitive tooth, and the oil having no affinity for moisture, forces the remaining moisture out of the cavity, and the gutta-percha adheres to the tooth. This is a valuable aid in treating deciduous teeth.—C. F. Ash, *Odontologist*.

*To Improve the Color of Pink Rubber.* Take a piece of absorbent cotton, saturate it with sulfuric acid, and rub it over the prosthetic rubber piece until it is black. Wash off every trace of the acid. The resulting color is a perfect imitation of the natural gums.—*LeLaboratoire et le Progres Dentaire* (*Dental Cosmos*).

*Saving Tooth Tissue.* The practitioner who has, in the past, delighted to call himself conservative, because he has preserved thin walls and weakened cusps, to break down later, and perhaps carry away with them sections of the all-important tooth root, or who has kept in the mouth of his patients, organs long past the stage of usefulness, and far advanced in their possibilities for harm, because it pleased his patients, has much to answer for.

If the gold inlay has done nothing more for us than to relieve us of the need for over-consideration of weak walls and undermined cusps, because of the time required to place an efficient substitute for them, it will have won a worthy place in our equipment.—H. W. Gillett, in *Journal of Allied Societies*.

*Emetin.* A few weeks ago Dr. A. J. McDonagh predicted that some enterprising drug company would place emetin on the market in a form for daily use by the public. This prediction has now come true. Patients suffering from pyorrhea carry a small bottle of the solution in their pocket or hand bag, and, as occasion demands, wind a little cotton wool on a toothpick, dip it into the solution and moisten the gums. One patient informed the writer that he could feel the gums just knit up around the necks of the teeth for several minutes after every application. There is going to be money in this game for some one. The simplicity of this treatment will take with the public. It will beat the alkaline mouth wash and the disinfectant mouth wash for dental caries as a money maker. The merits of this drug are being extolled in magazines and daily papers. The medical journals and the medical profession have fallen for it. They are injecting solutions of emetin into the arms of their patients with marvelous results on the pyorrhea. Histories of cases cured are being reported by them.

Before well-informed dentists can accept any such florid results they must have better evidence than that supplied by the public or physicians who do not know pyorrhea from gingivitis. This talk of injecting a few drops of emetin and thereby curing pyorrhea without removing the irritant is the statement of men who don't know much of pathology or practice, or have some ulterior motive to serve.

Dentistry must be exploited. The time is ripe. Every disease, from falling of the womb to loss of hair, is caused by pyorrhea, or septic roots. If a dentist ten years ago dared mention the fact that it was quite as important to see that the people had clean mouths as clean food he was promptly sat upon. It is up to dentists to steady the pendulum and not allow their profession to be stampeded and exploited.—Dr. Webster, in *Dominion Journal.*

*Amalgam Restoration.* Intelligently used, conscientiously shaped and fashioned, amalgam will produce as useful and satisfactory a means for the reproduction of lost parts of decayed teeth as any substance I know; and the man who uses it need not apologize to his patients for his work at any time. He may rest assured that his services to his patients are equal to those of many who make great claims concerning the superiority of gold; and he need have no compunction about charging the patient such fees as he may feel that his time and skill are worth.—H. L. Wheeler, in *Journal of Allied Societies.*

*Cause of Pyorrhea.* I am coming more and more to the conclusion that pyorrhea is a secondary manifestation. That is, it is a focus which is hematogenous in origin, just like the peri-dental abscess. As the bacteriological studies of pyorrhea improve, the percentage of streptococci in these reports rises higher and higher.

Pyorrhea is a disease of middle age—the period when foci are established, when sensitization to streptococci has taken place—when these foci manifest themselves in various parts of the body and the area in the gum adjacent to the tooth is one of these points of predilection. Pyorrhea thus stands as an embolic process in one of the end arteries of the gum, adjacent to the tooth. The reaction here is like the reaction in any other area of the body. There is produced a granulomatous mass which breaks down from lack of nutrition and establishes a sinus running along the tooth to the gingival margin. There is a great deal of wisdom in Doctor Kremer's remark that pyorrhea may be a barometer which measures the resistance or lack of resistance of the individual to streptococci.—Dr. H. L. Ulrich, in *Summary.*

*Annual Number of Dental Licentiates Inadequate.* The conviction seems to be growing that dental schools, notwithstanding their splendid success and increasing progress, are not educating enough men to recruit the ranks of the profession. It is estimated that there is a five per cent. loss each year on account of death and retirement; that about two thousand licenses should be granted each year to fill up the ranks. This allows nothing for the increase in population nor the increased demands for dental service. In 1913 twenty-one hundred students were graduated; of these but thirteen hundred and fifty, according to the report of the National Association of Dental Examiners, were licensed.

The proper recruiting of the profession is a matter of great moment to the public, and to boards of examiners as well as to dental schools. The public is demanding more and more dental service each year. The care of the state's dependents, the unfortunate inmates of penitentiaries, insane asylums, institutions for the feeble-minded, is a call to humanity that is going to do much to impress the necessity for dental services upon the public mind, and will, in the near future, tax the profession to its utmost, and it is the joint duty of these boards and schools to be ready to meet the call when it comes. In our efforts to "elevate the profession" let us not forget that the public has an interest that must be recognized. Let us acknowledge our responsibility by encouraging young men to prepare themselves for this service.

Every dentist should feel equally this duty, earnestly warn young men of the fatal mistake of entering upon this great work without the proper equipment, and urge the great need of well-trained men and their thorough preparation before entering the profession. Such a course will strengthen the efforts of the educator and make the duties of the state boards less difficult.—Dr. H. W. Morgan, in *Cosmos*.

*Need of Caution in Diagnosis.* We should be most guarded in using the word "cause" in relation to a possible focus of infection of a systemic disease. We should not with the evidence ordinarily before us say that that focus is the absolute cause of a systemic disturbance. There are so many factors to be taken into consideration that it would be best to refer patients to the family physician. As Doctor Kremer says, we must all get together. The oral surgeon has no right to diagnose a systemic condition of the patient and say that the mouth is related to it. Nor should the physician overstep his bounds and order teeth removed without consulting the oral surgeon.

I have personal knowledge of several cases of severe nervous breakdown, where the dentist in charge had pictured such dire results in these cases that it brought on illness requiring the patient to take to bed.

Rosenow and Ulrich state that systemic infection due to a focus of infection anywhere is probably of hematogenous or blood-borne origin. In the specific case of a devitalized tooth, where we are told that because of the lowered resistance, infection gains a foothold through the medium of the blood stream, can it be proven that the infection was not planted there at the time of that operation? Can any one of us say that when this operation is done under aseptic conditions and done thoroughly that these teeth show foci of infection at their root ends? What are we to do with those cases where the destruction of the dental pulp can not be avoided? Are we going to advise the sacrifice of that tooth? I can not agree with his viewpoint and should say that it is a most radical conclusion.

What we need most is to revise our methods and assure ourselves that we are doing this operation in a surgically clean manner. We are now reaping the results of our dirty, indifferent methods in the past, and I predict that where accurate data has been kept and the operation performed up to a high standard that the ratio will show us that as the laundries tell us, "It pays to keep clean."—W. H. MacNeil, in *Summary*.

*Origin of Alveolar Infection.* As there seems to be some little doubt in the minds of some as to the hematogenous origin of the streptococcus infection in the blind dental abscess, I would like to ask several questions. I believe that the answers that nearly every thinking dentist must give would do much to clear up this question.

1. Is it conceivable that a dentist, even using the worst asepsis, would often carry a streptococcus into the pulp canal of the tooth, even if we granted only ninety-five per cent. of blind dental abscesses as being streptococcus infections, would the dentist plant it that often possibly?

2. If we grant the above, how could we explain the presence of the streptococcus in the abscess in which the tooth has been devitalized as a result of traumatism, etc? Surely no dentist planted that!

3. Where also did the infection gain a footing in the so-called blind cystic abscess on a vital tooth if not by the way of the blood stream?

4. Is it possible for anyone to remove a pulp from a tooth by any known method without producing a hemorrhage in the apical space?

5. If so, what is the technic?

6. Can there be a hemorrhage in any tissue without producing an area of lowered resistance in that tissue likely to become infected?

In whatever way we may answer the above questions there is one matter that relates vitally to our policy as dentists to which we should give serious and immediate attention. We should ask ourselves whether in view of the tremendous percentage of abscesses occurring on the devitalized teeth are we justified in continuing the practice of devitalization? Isn't it better reasoning for us as dentists to discontinue the devitalization of teeth as a general practice until such time as these men who believe it a question of root canal technic can prove that such is the case, and until they can elaborate a method which can stand the test? Shall we continue what is certainly at the present time an indefensible practice at the expense of our patients' health and it may be of their life as well? Some arguments have been advanced that we who have been driven to believe in a rather radical extraction of devitalized teeth must prove our case. It would seem more reasonable in view of the preponderance of evidence on all sides that the advocates of devitalization should be compelled to prove their case first, unless perchance they feel what is properly a research problem should be worked out by the whole profession at the expense of the public.

In this connection I can not refrain from adding what I feel is the great lesson we must draw from our consideration of this question. I believe it will drive us eventually to a higher and better dentistry and incidentally to a proper consideration of the prevention of the necessity for devitalization of teeth. We should at once discontinue all systems of bridge work or crown construction which necessitate the devitalization of sound healthy teeth. Above all, I believe we should turn to the care of the young child's mouth and instead of teaching our students to be only repairers of teeth, we should make them real dentists, capable of taking proper care of the mouth and also capable of co-operating with others in the care of the whole body, that we may better approach our ideal of a normal mouth performing a normal function in a normal body.—Jay N. Pike, in *Summary.*

*Removing Rust from a No. 7 Chuck Handpiece.* If through neglect or other cause, a No. 7 handpiece becomes rusty inside, it should be immersed in paraffin oil for some hours. When the paraffin oil does not unrust the handpiece quickly enough, the part just behind the tip of the nose should be held over a Bunsen flame, care being taken not to make it too hot, and it should be plunged when thus warmed into the paraffin. This always releases the internal part.—*Australian Journal of Dentistry.*

*Reconstructing Gold Inlay Margins.* When a flaw in an inlay margin presents, the inlay is placed in the cavity, and after drying the surface of the inlay with a hot-air syringe and alcohol, a small piece of Alexander's gold is burnished with a warm burnisher to the surface of the inlay, and over the exposed cavity margin.

Then the whole is carefully removed from the cavity. This gold is incorporated with wax, which also serves as a flux; a small piece of 22-karat solder is placed where the gold is burnished to the inlay, and the case is set on the soldering frame. After the wax has burned out of the Alexander gold, the solder will flow and fill the remaining gold mesh. The inlay is then replaced in the cavity, burnished, and finished as usual.—G. H. W., *Northwestern Journal of Dentistry.*

*Aluminum-Vulcanite Plates.* An aluminum plate with teeth attached by vulcanite forms a very light denture, is comfortable to wear, and of little bulk. It is very well known, however, that after being for some time in use, the vulcanite is liable to separate from the aluminum. A method of obviating a breakdown from this cause would probably lead many practitioners who have abandoned the combination to try it afresh. When the case is ready for rubber packing, the cleaned aluminum surface is coated with a thin solution of rubber in chloroform, and over this when dry is carefully packed a layer of weighted rubber, and the further filling of the mold space completed with ordinary rubber. Those who have tried this method claim that the attachment between vulcanite and aluminum remains sound, and a stable union between the two materials is secured. A similar use of weighted rubber was some years ago found very serviceable with other metals in attaching teeth by vulcanite to ''wire bar'' lower cases; the resulting union was so very close and intimate that hardly anything short of filing or chiseling would effect their separation.—*Dental Record.*

*Improvement in Replantation.* Replanted teeth frequently become loose after some time owing to absorption of the apical root portion, and fall out, even though they may have remained firm for years. To prevent this absorption, Mayer provides a supply a small caps of soft 22-k. gold of from 2 to 3 mm. in height and of various widths, to which a fine pin of from 5 to 6 mm. length is soldered, which serves to retain the cap in place and as a partial root-canal filling. The method of application is as follows: After thoroughly cleaning the socket and removing all diseased portions of the root, the root-canal is enlarged from the apical foramen to allow insertion of the gold pin. A cap of suitable size is selected, and cemented firmly over the root, and its margins are burnished. In this way not only the large apical foramen, but also its accessory canaliculi are hermetically sealed-off against contact with the surrounding tissue, thus preventing the establishment of osteoclasts at the root-apex, and subsequent absorption. The writer has applied this method in three cases of replantation, the teeth becoming firm. Time alone will tell, of course, whether the osteoclasts may not find their way below the cap and start their destructive action, though this fear has not been confirmed by radiographs. Moreover, osteoclasts are found usually only in portions denuded of pericementum, hence it is important not to destroy this tissue before replanting a tooth. The writer fails to describe his method of filling the major portion of the root-canal in teeth to be replanted, which must, of course, be done by means of a permanent root-canal filling material, preferably gutta-percha or paraffin, or a combination of these.—Dr. Mayer, Mainz, Germany, in May *Cosmos*, p. 583.

*Recovering Mercury:* Place in the retort from two to three pounds of the old amalgam and apply the heat until the retort is brought to the required temperature, or about a cherry red, at which time the mercury will volatilize and pass as a gas through the pipe. When it reaches the second curve of the pipe, which is cooled by allowing water to drop upon it, the mercury deposits at this point in its metallic state and by a tap upon the pipe releases itself and flows into the vessel, which should be a bottle with the nose slipped over the outlet of the pipe. It is well to mention, at this point, that the operator should use care, as the fumes from the mercury are very poisonous and should not be inhaled. The mercury obtained may then be transferred to a vessel and treated with pure sulfuric acid. The fine particles of metal carried over with the gas will be dissolved and you will have "redistilled mercury," as sold by the supply man.—A. F. Linscot, in *Summary*.

*Emetin in Pyorrhea.* A patient reported at the office who had been suffering for years, and upon examination I found that pus could be compressed from each tooth socket. I proceeded to scale the local deposit from the teeth as best I could accomplish this for a first sitting and then injected emetin hydrochloride into all the pockets. The patient reported one week later and the characteristic pyorrhea pus odor had disappeared and I was surprised to notice that pus could be compressed from only two sockets. The character of this pus was entirely different from what it was the week previous. Instead of being a creamy consistency and color it had a white color and was mixed with considerable serum. These two pockets were treated by scaling the two teeth thoroughly followed by an injection of emetin, not only in these two pockets but into all the pockets which could be found. The next time the patient reported there was a slight discharge from these two pockets which I found was due to faulty scaling of the teeth. I proceeded to remove this deposit and the deposit which I had overlooked upon the other teeth and made a third injection. The next time the patient reported I gave a thorough prophylactic treatment and injected emetin into the pockets, which were then practically closed.

This patient reported three months later for prophylactic treatment and I could find no pus in her mouth.

Before the first injection of emetin was made I made a microscopical examination and found the coagulated amœbic parasite. Another examination of the discharge was made toward the close of the treatment and the parasites were entirely absent.—C. D. Lucas, in *Summary*.

*Alveolar Abscess and Systemic Infection.* It is of almost daily occurrence to have some layman visit us for the purpose of determining the relationship between his teeth and some particular disorder with which he may be afflicted. It is of almost equal frequency that some one of our good medical friends calls us to account for taking the position that every ill to which flesh is heir has a dental origin.

Within the week last past I have been brought to book by four physicians for this alleged heresy, and cautioned by them that "talking these things so freely with patients is likely to do much harm." These were all good friends of mine, and their remarks carried no rancor of a personal nature.

The very nature of the practice of medicine or any of its specialties fosters ultra-conservatism. The practitioner deals not only with pain and disease but carries responsibility for life itself, and if he hesitates to accept new doctrines, he has the best of reasons for it.

No one seriously disputes the influence of infected tonsils upon the heart and joints. Why? Because they are tonsils? Not at all. Simply because they are known to be favorable locations for the manufacture and distribution of those elements which affect joints and hearts unfavorably. No one would for a moment dispute the statement that these lethal elements are carried to heart and joints by way of the blood stream, and yet many of these same individuals hold up their hands and cry "heresy" when we assert that the same elements are generated at the root ends of teeth and are carried in the same manner to the same parts.

Furthermore, it is denied that the teeth are nearly so common a cause of infection as the tonsils by those who admit the possibility of both being involved. Here again it might be wise to consider the facts.

Prior to the introduction of the radiograph in dental surgery, we had no means of knowing root end conditions except as evidenced by swelling, soreness or loosening of teeth. We knew that we had two general types of abscesses affecting the teeth. The ordinary type with fistulous opening and the other type which we call "blind abscess" because it has no outward visible opening. These latter are extremely difficult of diagnosis except in acute stages when their presence is suspected by reason of pain or tenderness in the tooth upon pressure. By the aid of radiographic plates and films we can now make a diagnosis with accuracy in the majority of cases.

An examination of 269 radiographs of filled roots shows 211 defined abscesses, 55 cases of bone infiltration, and but three that were apparently free from pathological conditions.

From the advance pages of an article by Dr. Henry Ulrich upon this subject, I am permitted to quote that of 1,000 teeth examined by him seventy-one per cent. showed well-defined abscesses. Of the 269 cases referred to, there were 47 roots that were well and thoroughly filled, and 219 that were indifferently or badly done. It is of interest that the three cases that showed no pathology presented some of the worst examples of root filling that I ever examined. In the past six months we have made cultures from sixty cases of blind abscess and except in one instance we have developed streptococcic growths.

In the face of the foregoing facts, I think it perfectly safe to assert that teeth are a far more prolific source of infection than tonsils, as relates to adults. In routine examinations it is my custom to examine the tonsils, and while I find many cases that have to be referred to the throat specialist they are few and far between compared to teeth, as foci of infection. This statement does not obtain in the case of children who seem to be more prone to tonsillar infection than adults, and who are as much if not more frequently afflicted with cardiac infections than are their elders.

My investigations have not as yet carried me far enough to determine the part that abscesses of the deciduous teeth play, nor to prove the proportion of cases of this kind emanating from septic deciduous teeth and the tonsils.

Referring again to the condition of the roots of teeth as shown by radiographs, I am led to lay special emphasis for the necessity upon the part of my own profession, for more intimate knowledge of the consequences of devitalization of pulps, and the filling of root canals, and to urge them to take this matter up and study it from this new angle of vision, and satisfy themselves as to their right to do this operation in the face of the facts which they are sure to find. Show me the surgeon, if you can, who would for one moment consider performing any operation where the tabulated results show such an appalling percentage of failures, and I will show you a man who would be read out of his profession as soon as the facts became known to his fellow practitioners.

This is a showing that ought to deter every thinking man from practicing pulp devitalization and root filling except it be under the most favorable circumstances and conditions, and then only with the full knowledge and consent of the patient who had been advised of the possibilities that may later confront him.—Extract from article by F. B. Kramer in April *Summary.*

*Dr. Buckley's Desensitizing Paste.* Allow me to give you a brief history of my experience with Buckley's desensitizing paste. I used it for some time without any disagreeable results, but of late, and with the same material I at first used, I have had many cases of pulp hyperemia and a few cases of devitalization. I would not think of using it in the cavity very near a pulp, for it is almost sure to devitalize the tooth when so used, but I have had not a few cases of severe pulp irritation—some transient and some persistent—even when it was used in shallow cavities.

There is no question as to its efficacy in obtunding dentin in any cavity, but it seems almost impossible to determine when it is safe to use it and when not. I believe thoroughly in the honesty and good intent of Dr. Buckley, but I fear that his statement in regard to the impossibility of the paste causing devitalization was rather premature.

I think you will find others have had a similar experience to mine, and I write this as a warning to those who are experimenting with this material, to use it with great caution. I was very enthusiastic about it at first and used it a great deal, but I must confess that I am now somewhat disappointed in it. I have never had so many cases of pulp irritation on hand as I have had lately, and am thoroughly convinced that this is due solely to the use of the paste.

Dr. Buckley has done much for dentistry in teaching us the advantages of the formocresol treatment, and he deserves unlimited credit for that discovery or propaganda, but I fear he has made a mistake in claiming so much for the desensitizing paste.—Dr. A. R. Starr, writing to the *Dental Cosmos,* for May 1915.

*Army Dental Surgeons.* Of the ninety-two candidates who presented themselves for the past three examinations, eleven failed physically, eight withdrew, forty-four failed either in mental or clinical work, and twenty-nine passed. The actual length of time spent in civil practice by these twenty-nine averaged two years and eight months. The law requires that a man be not over twenty-seven years of age when entering the dental corps. At the last examination held there were ten successful candidates, but it was possible to give contracts to only seven of them, as the act of Congress making appropriations for the fiscal year ending on June 30, 1915, made provision for only forty acting dental surgeons. The report of the Adjutant-general, United States army, June 30, 1914, gives the strength of the army as 92,877 enlisted men and 4,883 officers. The law allows one dental surgeon to each thousand men of the actual enlisted force of the army, so that, if the corps were filled on the basis of the 1914 report, it would consist of ninety-two dental surgeons, sixty of whom can become first lieutenant dental surgeons, the balance remaining acting dental surgeons until a vacancy occurs.—Dr. S. D. Boak, Dental Surgeon U. S. Army, in *Dental Cosmos.*

*Pyorrhea a Cause of Serumal Calculus.* It seems to me, that the only influence pus infection—pyorrhea—has in connection with the disease "interstitial gingivitis" is that it is a medium through which the absorbed alveolar process is conveyed from the process and deposited upon the roots of the tooth, and is called by the dental profession "serumal deposits."—E. S. Talbott, in *Cosmos.*

*Emetin and Pyorrhea.* Barrett says that "None of those who have been engaged in the study have held or now believe that all cases of pyorrhea are due primarily or alone to the presence of the oral endamœba." Again, he says, "After using the emetin treatment the patients invariably feel better, and there is unmistakable improvement; yet prolonged treatment with emetin does not effect a complete cure." After all, he must resort to the iodin treatment, for he says, "There is obviously something more to be done. . . . As a matter of fact, it has been found that a 1 per cent. solution of iodin in normal salt solution has usually completely removed the persisting condition." Hence it will be seen that he does not consider that emetin is a specific for pyorrhea. In order to get good results the deposit must be removed from the roots of the teeth. He lays considerable stress upon this procedure. If the disease is due to the amœba, and it can be cured by the application of emetin, why remove the deposits? My experience has been that by removing the deposits alone the pus infection nearly or quite ceases. The presence of pus is a splendid diagnostic sign that the deposits are not all removed.—E. S. Talbot, in May *Cosmos*.

*Treatment of Interstitial Gingivitis.* The first thing to do in the treatment of the mouth is to place the mouth in a presentable condition, by the application of iodin about the gums, teeth, and mucous membrane. The number of visits and applications will depend upon the condition of the mouth and the fastidiousness of the operator. No operator is justified in placing his hands in or spending much time examining a dirty mouth.

Give the patient a gum-wash and a gum-massage brush, and request him to use both vigorously until presentable for operating. The gum-massage brush should be made of unbleached bristles to obtain stiffness. Remove all irritants, both local and constitutional. See that the patient is in good health. Place the eliminating organs in a normal condition. Deplete the gums and alveolar process of stagnant blood; stimulate the bloodvessels and nerves to a healthy action. This may be accomplished by vigorous gum massage and astringent gum-washes or by vacuum treatment, or both. This treatment will improve the health of the tissues, but will not completely cure the disease.—E. S. Talbott, in *Cosmos*.

*Taking Modeling Compound Impressions.* We shall now take our impressions, according to this method, beginning with the upper, after having first relieved any congested condition of the mouth by means of a solution of zinc chloride or some other safe astringent. We select a modeling compound of "medium heat" which will take a sharp, clean impression and which will not cake on the surface nor stick messily to the model when we seek to remove it. We select a cup that permits a good body of the impression material to stand between it and the mouth. The rim of this cup should be rather low in front and slope to a greater height over the ends that are to cover the condoloid prominences. The compound is now softened placed in the cup and carried into place in the mouth, worked into place and held firmly until it hardens.

It is then removed and thoroughly chilled with cold water. Now, with a heated knife blade, those parts of the impression that extend over the velum and also the edges of the rim that have been forced too high over the buccal and labial aspects of the alveolus are trimmed away. Hot water is now poured over the surface of the impression, so that it may be softened evenly to a depth of about 3-32 in. It is now replaced in the mouth and forced home with sufficient pressure to insure the compression of all the soft places in the gums, and to accurately reproduce the various inequalities of their surfaces, during which operation the upper lip of the patient should be pressed against the softened compound beneath it in order to compress the membranous tissues around the labial surface of the ridge.

The impression is held in place for several minutes, until it has cooled, when it is again removed and chilled with cold water. It should be noted that our first impression is, in fact, an accurately moulded impression cup formed for the purpose of affording a foundation of modelling compound of progressively increasing hardness with which to drive its softened surface into exact, forcible contact with the tissues involved. It will also be noted that the volume of the stratum of softened modeling compound is markedly less than that of the entire body of compound in the impression cup, so that the proportion of contraction has thus been reduced to a point where it almost exactly counterbalances the expansion of the plaster model.—Wm. W. Atkinson, in *N. J. Journal.*

*To Remove Amalgam from the Fingers.* After kneading and squeezing out excess mercury, the thumb and index finger are more or less coated. It is then hard to work with crown gold without tarnishing same. Clean the fingers by heating a piece of bees wax slightly, and press firmly between coated fingers.—P. J. Pecau, in *Review.*

*To Remove Ink Stains.* When the finger is stained with ink and you have no sand soap in the office, just take a match, moisten slightly and rub over the discolored parts.—F. S. Dilger, in *Review.*

*The Sterilization of Instruments.* One is safe in sterilizing instruments by boiling them, but there are some drawbacks to this method which hinder its application. One is rusting and thereby marring the appearance of the instruments.

A very efficacious method I use in my office for my forceps, and it can be used for all other instruments, is one pint of tincture of green soap with four grains of bichlorid of mercury.

Procure a large glass jar, Mason or glass-covered one, with sufficient depth that the instruments can stand on end without interfering with cover. Put quantity of water in to cover two inches or more of the instruments and add to this one ounce of the solution.

This will not tarnish the instruments however long they remain in it.—Dudley Dean Bayless, in *Review.*

